# The furan­osteroid viridiol

**DOI:** 10.1107/S1600536813005606

**Published:** 2013-03-02

**Authors:** Pierre F. Andersson, Anders Broberg, Daniel Lundberg

**Affiliations:** a Department of Chemistry, Uppsala BioCenter, P.O. Box 7015, Swedish University of Agricultural Sciences, SE-750 07 Uppsala, Sweden

## Abstract

The asymmetric unit of the title compound, C_20_H_18_O_6_ (systematic name: 1β,3β-dihy­droxy-2β-meth­oxyfuro[4′,3′,2′:4,5,6]-18-norandrosta-8,11,13-triene-7,17-dione), a dihydro derivative of the fungal steroid viridin, contains two mol­ecules with similar conformations. The rings bearing the hy­droxy groups adopt boat conformations. The absolute structure was assigned based on the known chirality of a precursor compound. In the crystal, mol­ecules are linked by O—H⋯O hydrogen bonds, generating a three-dimensional network and weak C—H⋯O inter­actions consolidate the packing.

## Related literature

For background to fungal metabolites, see: Brian & McGowan (1945 ▶); Moffatt *et al.* (1969 ▶); Jones & Hancock (1987 ▶); Hanson (1995 ▶); Cross *et al.* (1995 ▶); Przybyl (2002 ▶); Smith *et al.* (2009 ▶); Andersson *et al.* (2010 ▶); Queloz *et al.* (2011 ▶); Andersson (2012 ▶); Andersson *et al.* (2012 ▶, 2013 ▶). For related structures, see: Neidle *et al.* (1972 ▶); Lang *et al.* (2009 ▶). For other characterization methods, see: Brian *et al.* (1957 ▶); Aldridge *et al.* (1975 ▶); Blight & Grove (1986 ▶). For background to the assignment of the absolute structure of the title compound, see: MacMillan *et al.* (1972 ▶); Harrison (1990 ▶); Dewick (2002 ▶); Wipf & Kerekes (2003 ▶); Flack & Bernardinelli (2000 ▶).
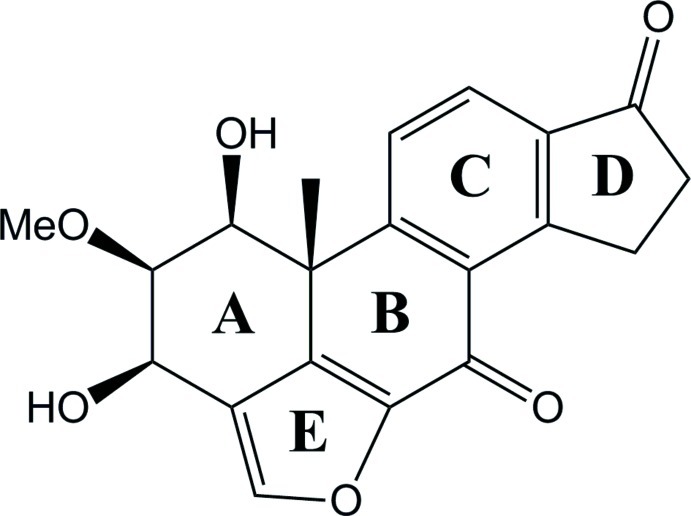


## Experimental

### 

#### Crystal data

C_20_H_18_O_6_
*M* *_r_* = 354.34
Orthorhombic, 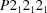

*a* = 6.8285 (2) Å
*b* = 20.1939 (6) Å
*c* = 22.4344 (6) Å
*V* = 3093.57 (15) Å^3^
*Z* = 8
Mo *K*α radiation
μ = 0.11 mm^−1^
*T* = 93 K
0.3 × 0.25 × 0.2 mm

#### Data collection

Oxford Diffraction XcaliburIII Sapphire-3 CCD diffractometer
Absorption correction: multi-scan (*CrysAlis RED*; Oxford Diffraction, 2007 ▶) *T*_min_ = 0.891, *T*_max_ = 1.000
26916 measured reflections
4874 independent reflections
3294 reflections with *I* > 2σ(*I*)
*R*_int_ = 0.085

#### Refinement

*R*[*F* ^2^ > 2σ(*F* ^2^)] = 0.049
*wR*(*F* ^2^) = 0.089
*S* = 0.91
4874 reflections
485 parameters
H atoms treated by a mixture of independent and constrained refinement
Δρ_max_ = 0.33 e Å^−3^
Δρ_min_ = −0.25 e Å^−3^

### 

Data collection: *CrysAlis CCD* (Oxford Diffraction, 2007 ▶); cell refinement: *CrysAlis RED* (Oxford Diffraction, 2007 ▶); data reduction: *CrysAlis RED*; program(s) used to solve structure: *SHELXD* (Sheldrick, 2008 ▶); program(s) used to refine structure: *SHELXL97* (Sheldrick, 2008 ▶); molecular graphics: *SHELXL* (Sheldrick, 2008 ▶); software used to prepare material for publication: *DIAMOND* (Brandenburg, 2001 ▶).

## Supplementary Material

Crystal structure: contains datablock(s) I, global. DOI: 10.1107/S1600536813005606/hb7028sup1.cif

Structure factors: contains datablock(s) I. DOI: 10.1107/S1600536813005606/hb7028Isup2.hkl

Supplementary material file. DOI: 10.1107/S1600536813005606/hb7028Isup3.cml

Additional supplementary materials:  crystallographic information; 3D view; checkCIF report

## Figures and Tables

**Table 1:** Hydrogen-bond geometry (Å, °)

*D*—H⋯*A*	*D*—H	H⋯*A*	*D*⋯*A*	*D*—H⋯*A*
O19*A*—H19*A*⋯O19*B* ^i^	0.85 (3)	2.08 (3)	2.886 (3)	160 (3)
O19*B*—H19*B*⋯O20*A* ^ii^	0.83 (3)	2.30 (3)	3.002 (3)	143 (3)
O20*A*—H20*A*⋯O25*B* ^iii^	0.91 (3)	1.85 (3)	2.717 (3)	160 (3)
O20*B*—H20*B*⋯O24*A* ^iv^	0.81 (3)	2.05 (3)	2.842 (3)	166 (3)
C2*A*—H2*A*⋯O23*B* ^v^	1.00	2.45	3.325 (3)	146
C2*B*—H2*B*⋯O23*A*	1.00	2.38	3.295 (3)	151
C11*A*—H11*A*⋯O19*A*	0.95	2.43	3.084 (3)	126
C11*B*—H11*B*⋯O19*B*	0.95	2.58	3.230 (3)	126
C18*A*—H18*A*⋯O20*A*	0.98	2.38	3.227 (3)	145
C18*B*—H18*D*⋯O20*B*	0.98	2.39	3.241 (4)	145
C21*A*—H21*A*⋯O24*A* ^vi^	0.95	2.37	3.282 (3)	162
C21*B*—H21*B*⋯O24*B* ^vii^	0.95	2.25	3.180 (3)	167

Symmetry codes: (i) 
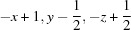
; (ii) 
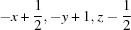
; (iii) 
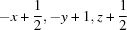
; (iv) 
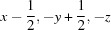
; (v) 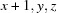; (vi) 
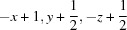
; (vii) 
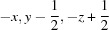
.

**Fig. 1.**
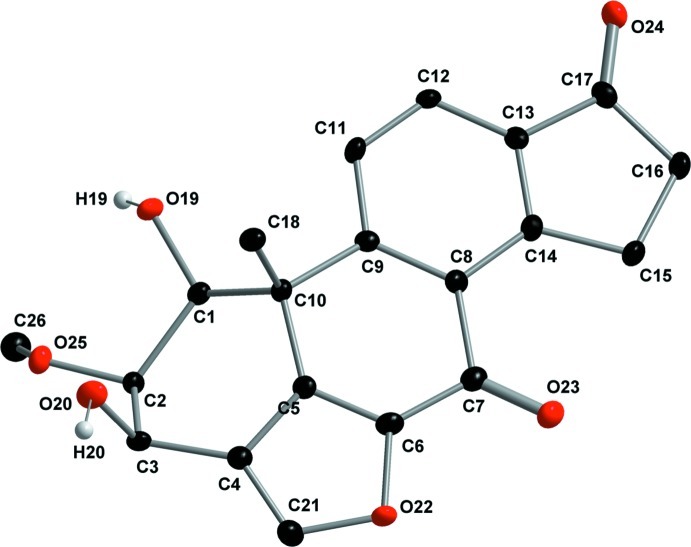
Labelling of (I) follows the system used previously (Aldridge et al., 1975). H atoms on alkyl and aryl carbons have been removed for clarity. Displacement ellipoids are set at 50%.

**Fig. 2.**
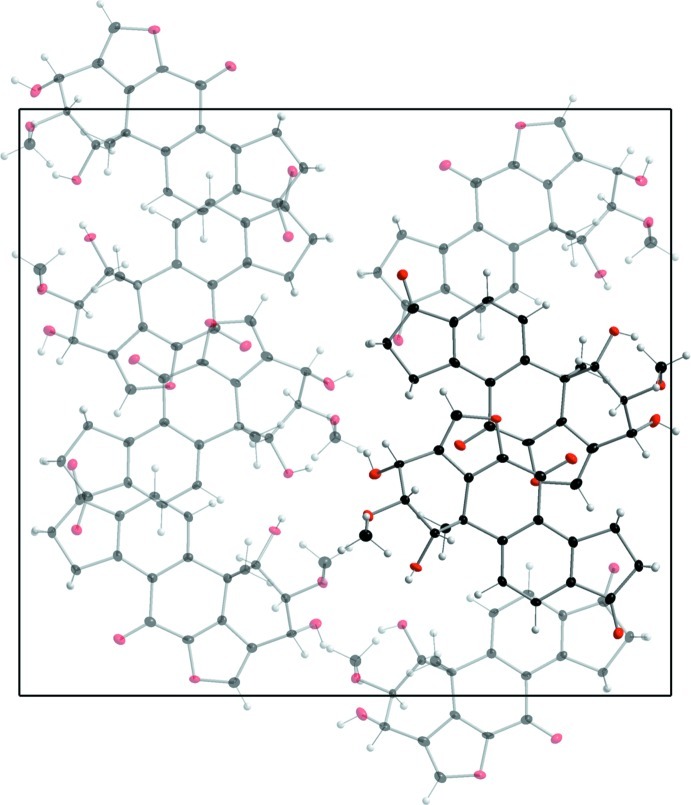
Unit cell packing of (I), viewed along the a axis.

**Fig. 3.**
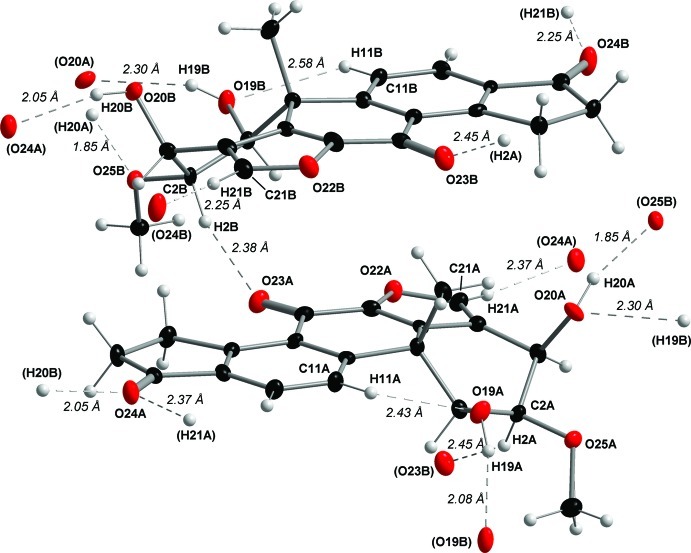
One intramolecular and many intermolecular hydrogen bonds in the range 1.85–2.58 Å are present in (I), including both conventional (O–H···O) and non-conventional (C–H···O) ones.

**Fig. 4.**
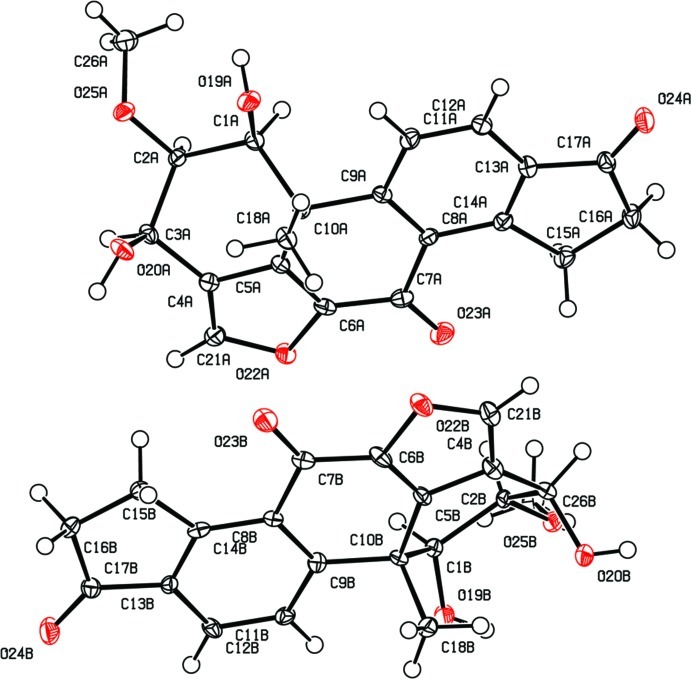
ORTEP plot of (I).

## supplementary crystallographic information

### Comment

The disease known as dieback of ash on European ash (*Fraxinus excelsior*) was first observed in Poland in 1995, but has spread rapidly over most of the European subcontinent (Przybyl, 2002). During studies of the secondary metabolite production of the fungus *Hymenoscyphus pseudoalbidus*, the pathogen responsible for dieback of ash (Queloz *et al.*, 2011), a number of steroidal compounds have been isolated (Andersson *et al.*, 2010; Andersson *et al.* 2013; Andersson *et al.* 2012). These compounds belong to a family of fungal steroids (Hanson, 1995), of which some have been shown to have interesting bioactivities (Cross *et al.* 1995; Smith *et al.* 2009; Andersson 2012).

The first reported compound of this family was viridin (Brian & McGowan, 1945), with its crystal structure published nearly 30 years later (Neidle *et al.*, 1972). The absolute structures of a few other members have been reported through successful osmylation (Lang *et al.* 2009). Other members of the same family have also been characterized through other methods than crystallography, including wortmannin (Brian *et al.*, 1957), demethoxyviridin and demethoxyviridiol (Aldridge *et al.*, 1975), and virone and wortmannolone (Blight & Grove, 1986). The phytotoxin viridiol (Moffatt *et al.*, 1969), which has also been suggested to be part of the pathogenicity of *H. pseudoalbidus* (Andersson *et al.* 2010), can be produced in *Gliocladium virens* from viridin (Jones & Hancock, 1987). The previously reported absolute configuration of these compounds are based on the evidence of their steroidal origin i. e. the configuration of the C10 carbon is based on lanosterol (Dewick, 2002; Harrison, 1990). Here, we present the crystal structure of viridiol (I) confirming the previously presented structure, both relative and absolute (MacMillan *et al.*, 1972; Moffatt *et al.*, 1969; Wipf & Kerekes, 2003) (Scheme 1, Fig. 1)

Compound (I) crystallizes in the orthorhombic space group *P*2_1_2_1_2_1_ (No. 19), with two crystallographically independent viridiol molecules with a total of eight in the unit cell. (Fig. 2) The nearly flat furanosteroid skeleton lies in the *bc* plane, with the A ring and its methoxy group bending away from the plane (Fig. 1). In the crystal, O—H···O and C—H···O interactions link the molecules (Table 1, Fig. 3).

### Experimental

The viridiol containing fraction from a previous study (Andersson *et al.*, 2013) was subjected to rotatory evaporation, which lead to crystal formation. The crystals, too small for crystallography, were harvested by filtration and dried (approx. 3 mg). The crystals were subsequently dissolved in 80 °C toluene (4 ml) in a 5 ml test tube. The solution was left at room temperature and the toluene was evaporated slowly by a gentle stream of nitrogen gas. Large enough crystals formed at the bottom of the test tube after stepwise precipitation of impurities on the inner test tube wall. A colourless block was mounted on a glass capillary and a data set was measured under cold conditions (93 K).

### Refinement

After initial integration, the furanosteroid backbone was found through refinements using *SHELXD*. After additional cycles in *SHELXL*, the remaining atoms were found. No restraints were applied to the carbon skeleton. All non-H atoms were refined anisotropically. Hydrogen atoms on carbons were refined as riding on their respective carbon, while the two hydroxy hydrogen were fully refined. In the absence of any significant anomalous scattering, the Flack parameter was indeterminate (Flack & Bernardinelli, 2000). Hence, the Friedel equivalents were merged prior to the final refinements, and the absolute structure was set by reference to the known chirality of the pathway for the previously reported precursor lanosterol (Dewick, 2002; Harrison, 1990) (Fig. 4).

### Crystal data

**Table:**

C_20_H_18_O_6_	*F*(000) = 1488
*M**_r_* = 354.34	*D*_x_ = 1.522 Mg m^−^^3^
Orthorhombic, *P*2_1_2_1_2_1_	Mo *K*α radiation, λ = 0.71073 Å
Hall symbol: P 2ac 2ab	Cell parameters from 5568 reflections
*a* = 6.8285 (2) Å	θ = 2.9–32.3°
*b* = 20.1939 (6) Å	µ = 0.11 mm^−^^1^
*c* = 22.4344 (6) Å	*T* = 93 K
*V* = 3093.57 (15) Å^3^	Block, colourless
*Z* = 8	0.3 × 0.25 × 0.2 mm

### Data collection

**Table:**

Oxford Diffraction XcaliburIII Sapphire-3 CCD diffractometer	4874 independent reflections
Radiation source: fine-focus sealed tube	3294 reflections with *I* > 2σ(*I*)
Graphite monochromator	*R*_int_ = 0.085
Detector resolution: 16.5467 pixels mm^-1^	θ_max_ = 29.6°, θ_min_ = 3.1°
ω scans at different φ	*h* = −9→8
Absorption correction: multi-scan (*CrysAlis RED*; Oxford Diffraction, 2007)	*k* = −27→28
*T*_min_ = 0.891, *T*_max_ = 1.000	*l* = −31→27
26916 measured reflections	

### Refinement

**Table:**

Refinement on *F*^2^	Secondary atom site location: difference Fourier map
Least-squares matrix: full	Hydrogen site location: inferred from neighbouring sites
*R*[*F*^2^ > 2σ(*F*^2^)] = 0.049	H atoms treated by a mixture of independent and constrained refinement
*wR*(*F*^2^) = 0.089	*w* = 1/[σ^2^(*F*_o_^2^) + (0.0383*P*)^2^] where *P* = (*F*_o_^2^ + 2*F*_c_^2^)/3
*S* = 0.91	(Δ/σ)_max_ < 0.001
4874 reflections	Δρ_max_ = 0.33 e Å^−^^3^
485 parameters	Δρ_min_ = −0.25 e Å^−^^3^
0 restraints	Absolute structure: syn
Primary atom site location: structure-invariant direct methods	

### Special details

**Table:**

Geometry. All e.s.d.'s (except the e.s.d. in the dihedral angle between two l.s. planes) are estimated using the full covariance matrix. The cell e.s.d.'s are taken into account individually in the estimation of e.s.d.'s in distances, angles and torsion angles; correlations between e.s.d.'s in cell parameters are only used when they are defined by crystal symmetry. An approximate (isotropic) treatment of cell e.s.d.'s is used for estimating e.s.d.'s involving l.s. planes.
Refinement. Refinement of *F*^2^ against ALL reflections. The weighted *R*-factor *wR* and goodness of fit *S* are based on *F*^2^, conventional *R*-factors *R* are based on *F*, with *F* set to zero for negative *F*^2^. The threshold expression of *F*^2^ > σ(*F*^2^) is used only for calculating *R*-factors(gt) *etc*. and is not relevant to the choice of reflections for refinement. *R*-factors based on *F*^2^ are statistically about twice as large as those based on *F*, and *R*- factors based on ALL data will be even larger.

### Fractional atomic coordinates and isotropic or equivalent isotropic displacement parameters (Å^2^)

**Table:**

	*x*	*y*	*z*	*U*_iso_*/*U*_eq_	
C1A	0.6537 (4)	0.27709 (14)	0.36272 (12)	0.0138 (6)	
H1A	0.7696	0.2648	0.3378	0.017*	
C2A	0.7233 (4)	0.33366 (13)	0.40850 (12)	0.0126 (6)	
H2A	0.8510	0.3506	0.3928	0.015*	
C3A	0.5943 (4)	0.39445 (13)	0.41746 (12)	0.0133 (6)	
H3A	0.6728	0.4296	0.4379	0.016*	
C4A	0.5392 (4)	0.41850 (13)	0.35652 (12)	0.0130 (6)	
C5A	0.5063 (4)	0.37163 (12)	0.31066 (11)	0.0112 (5)	
C6A	0.4956 (5)	0.40461 (12)	0.25876 (11)	0.0144 (6)	
C7A	0.4926 (4)	0.37424 (13)	0.20055 (11)	0.0142 (6)	
C8A	0.4930 (5)	0.29928 (12)	0.20388 (11)	0.0129 (6)	
C9A	0.4948 (4)	0.26436 (12)	0.25865 (11)	0.0111 (6)	
C10A	0.4855 (5)	0.29948 (12)	0.31901 (11)	0.0125 (6)	
C11A	0.4926 (5)	0.19481 (13)	0.25915 (11)	0.0154 (6)	
H11A	0.4941	0.1721	0.2962	0.019*	
C12A	0.4883 (5)	0.15934 (13)	0.20748 (11)	0.0142 (6)	
H12A	0.4869	0.1123	0.2082	0.017*	
C13A	0.4860 (5)	0.19347 (13)	0.15357 (11)	0.0128 (6)	
C14A	0.4907 (5)	0.26182 (13)	0.15062 (11)	0.0128 (6)	
C15A	0.4908 (5)	0.28556 (13)	0.08641 (11)	0.0170 (6)	
H15A	0.6103	0.3115	0.0776	0.020*	
H15B	0.3744	0.3133	0.0781	0.020*	
C16A	0.4857 (5)	0.22141 (13)	0.04961 (11)	0.0169 (6)	
H16A	0.3696	0.2209	0.0232	0.020*	
H16B	0.6048	0.2177	0.0247	0.020*	
C17A	0.4762 (4)	0.16537 (14)	0.09340 (11)	0.0144 (6)	
C18A	0.2815 (4)	0.28525 (15)	0.34602 (12)	0.0152 (6)	
H18A	0.2704	0.3073	0.3848	0.023*	
H18B	0.2650	0.2374	0.3512	0.023*	
H18C	0.1798	0.3021	0.3192	0.023*	
O19A	0.5889 (3)	0.21883 (10)	0.39170 (9)	0.0183 (5)	
H19A	0.690 (5)	0.1979 (16)	0.4026 (14)	0.027*	
O20A	0.4303 (3)	0.37778 (10)	0.45422 (9)	0.0175 (5)	
H20A	0.360 (5)	0.4134 (16)	0.4665 (13)	0.026*	
C21A	0.5428 (4)	0.47768 (14)	0.32940 (12)	0.0169 (7)	
H21A	0.5620	0.5185	0.3495	0.020*	
O22A	0.5152 (3)	0.47204 (8)	0.26879 (7)	0.0163 (4)	
O23A	0.4968 (3)	0.40411 (9)	0.15318 (8)	0.0202 (5)	
O24A	0.4658 (3)	0.10611 (9)	0.08101 (8)	0.0197 (5)	
O25A	0.7644 (3)	0.30680 (10)	0.46491 (8)	0.0155 (5)	
C26A	0.9470 (5)	0.27292 (15)	0.46869 (13)	0.0198 (7)	
H26A	0.9599	0.2425	0.4349	0.030*	
H26B	0.9525	0.2478	0.5060	0.030*	
H26C	1.0542	0.3052	0.4678	0.030*	
C1B	0.1585 (4)	0.56087 (14)	0.11712 (12)	0.0138 (6)	
H1B	0.2773	0.5707	0.1416	0.017*	
C2B	0.2209 (4)	0.50356 (13)	0.07249 (11)	0.0122 (6)	
H2B	0.3410	0.4826	0.0897	0.015*	
C3B	0.0753 (4)	0.44728 (14)	0.06042 (12)	0.0142 (6)	
H3B	0.1451	0.4112	0.0385	0.017*	
C4B	0.0186 (5)	0.42186 (13)	0.12046 (12)	0.0148 (6)	
C5B	0.0000 (5)	0.46678 (12)	0.16840 (11)	0.0122 (6)	
C6B	−0.0095 (5)	0.43195 (12)	0.21909 (12)	0.0153 (6)	
C7B	0.0047 (5)	0.45916 (13)	0.27823 (12)	0.0147 (6)	
C8B	0.0128 (5)	0.53407 (12)	0.27772 (11)	0.0131 (6)	
C9B	0.0140 (4)	0.57193 (13)	0.22427 (11)	0.0124 (6)	
C10B	−0.0063 (5)	0.54023 (12)	0.16285 (11)	0.0122 (6)	
C11B	0.0197 (4)	0.64112 (12)	0.22764 (11)	0.0130 (6)	
H11B	0.0221	0.6661	0.1917	0.016*	
C12B	0.0220 (5)	0.67419 (13)	0.28128 (11)	0.0142 (6)	
H12B	0.0244	0.7212	0.2827	0.017*	
C13B	0.0207 (4)	0.63669 (13)	0.33360 (11)	0.0111 (6)	
C14B	0.0183 (4)	0.56769 (12)	0.33227 (11)	0.0126 (6)	
C15B	0.0213 (5)	0.53980 (13)	0.39498 (11)	0.0143 (6)	
H15C	0.1383	0.5116	0.4011	0.017*	
H15D	−0.0974	0.5130	0.4027	0.017*	
C16B	0.0272 (5)	0.60024 (13)	0.43590 (11)	0.0152 (6)	
H16C	−0.0855	0.5998	0.4636	0.018*	
H16D	0.1496	0.6006	0.4596	0.018*	
C17B	0.0180 (5)	0.66011 (13)	0.39581 (11)	0.0148 (6)	
C18B	−0.2121 (5)	0.55864 (15)	0.13852 (13)	0.0175 (7)	
H18D	−0.2304	0.5386	0.0991	0.026*	
H18E	−0.2231	0.6069	0.1353	0.026*	
H18F	−0.3128	0.5420	0.1658	0.026*	
O19B	0.1100 (3)	0.62125 (10)	0.08747 (9)	0.0187 (5)	
H19B	0.052 (5)	0.6121 (16)	0.0560 (14)	0.028*	
O20B	−0.0878 (3)	0.46870 (10)	0.02504 (9)	0.0194 (5)	
H20B	−0.090 (5)	0.4507 (15)	−0.0072 (14)	0.029*	
C21B	0.0133 (5)	0.36090 (14)	0.14567 (12)	0.0180 (6)	
H21B	0.0199	0.3206	0.1240	0.022*	
O22B	−0.0029 (3)	0.36481 (8)	0.20658 (8)	0.0182 (4)	
O23B	0.0138 (4)	0.42681 (9)	0.32411 (8)	0.0211 (5)	
O24B	0.0124 (4)	0.71751 (9)	0.41237 (8)	0.0244 (5)	
O25B	0.2759 (3)	0.53000 (9)	0.01613 (8)	0.0145 (4)	
C26B	0.4663 (4)	0.55951 (14)	0.01558 (12)	0.0165 (6)	
H26D	0.4740	0.5934	0.0468	0.025*	
H26E	0.4899	0.5800	−0.0233	0.025*	
H26F	0.5654	0.5254	0.0229	0.025*	

### Atomic displacement parameters (Å^2^)

**Table:**

	*U*^11^	*U*^22^	*U*^33^	*U*^12^	*U*^13^	*U*^23^
C1A	0.0182 (16)	0.0109 (15)	0.0122 (14)	0.0002 (12)	0.0016 (12)	0.0007 (12)
C2A	0.0151 (16)	0.0110 (14)	0.0118 (15)	0.0000 (12)	−0.0030 (12)	0.0008 (12)
C3A	0.0185 (16)	0.0079 (14)	0.0136 (14)	−0.0002 (12)	0.0016 (12)	−0.0013 (12)
C4A	0.0125 (16)	0.0121 (14)	0.0145 (14)	0.0004 (12)	−0.0001 (12)	−0.0001 (11)
C5A	0.0091 (14)	0.0125 (13)	0.0119 (13)	0.0005 (13)	0.0025 (13)	−0.0004 (10)
C6A	0.0152 (15)	0.0095 (14)	0.0185 (14)	0.0027 (14)	0.0001 (14)	0.0001 (11)
C7A	0.0112 (15)	0.0141 (13)	0.0174 (14)	−0.0001 (14)	−0.0007 (14)	0.0025 (12)
C8A	0.0134 (15)	0.0120 (13)	0.0134 (13)	0.0012 (14)	−0.0038 (13)	0.0007 (11)
C9A	0.0083 (14)	0.0108 (13)	0.0141 (13)	−0.0001 (13)	0.0005 (13)	−0.0004 (10)
C10A	0.0162 (16)	0.0113 (13)	0.0101 (12)	−0.0001 (14)	0.0004 (13)	0.0007 (10)
C11A	0.0211 (16)	0.0144 (14)	0.0108 (13)	−0.0001 (15)	0.0002 (14)	0.0033 (11)
C12A	0.0188 (16)	0.0085 (12)	0.0152 (14)	−0.0021 (14)	−0.0013 (14)	0.0011 (11)
C13A	0.0117 (15)	0.0142 (13)	0.0125 (13)	−0.0026 (14)	0.0023 (13)	−0.0013 (11)
C14A	0.0109 (15)	0.0159 (14)	0.0116 (13)	0.0009 (14)	0.0004 (13)	0.0017 (10)
C15A	0.0234 (17)	0.0141 (14)	0.0134 (13)	−0.0011 (15)	−0.0019 (14)	0.0036 (11)
C16A	0.0231 (17)	0.0176 (14)	0.0099 (13)	−0.0016 (15)	0.0023 (14)	0.0009 (11)
C17A	0.0124 (16)	0.0169 (14)	0.0139 (14)	0.0008 (14)	0.0010 (13)	−0.0039 (12)
C18A	0.0186 (16)	0.0125 (15)	0.0145 (15)	−0.0011 (13)	0.0036 (12)	−0.0005 (12)
O19A	0.0266 (14)	0.0097 (11)	0.0186 (11)	−0.0012 (10)	−0.0061 (9)	0.0043 (9)
O20A	0.0188 (12)	0.0145 (11)	0.0191 (11)	0.0039 (9)	0.0067 (9)	−0.0023 (9)
C21A	0.0189 (18)	0.0146 (15)	0.0172 (15)	0.0015 (13)	−0.0044 (13)	−0.0010 (12)
O22A	0.0256 (12)	0.0077 (9)	0.0156 (10)	−0.0006 (10)	−0.0014 (10)	0.0008 (7)
O23A	0.0269 (12)	0.0153 (10)	0.0185 (10)	−0.0001 (11)	−0.0040 (11)	0.0042 (8)
O24A	0.0285 (13)	0.0172 (11)	0.0134 (10)	−0.0004 (10)	0.0014 (9)	−0.0024 (8)
O25A	0.0172 (12)	0.0160 (11)	0.0133 (10)	0.0024 (9)	−0.0013 (8)	0.0035 (9)
C26A	0.0192 (18)	0.0183 (16)	0.0220 (16)	0.0018 (14)	−0.0027 (13)	0.0008 (13)
C1B	0.0179 (17)	0.0129 (16)	0.0106 (14)	0.0027 (13)	−0.0007 (12)	−0.0002 (12)
C2B	0.0188 (16)	0.0107 (14)	0.0070 (13)	0.0024 (12)	−0.0005 (12)	0.0002 (11)
C3B	0.0170 (17)	0.0102 (14)	0.0153 (15)	0.0035 (13)	−0.0002 (12)	−0.0018 (12)
C4B	0.0114 (15)	0.0133 (14)	0.0196 (15)	−0.0031 (14)	0.0018 (13)	−0.0041 (11)
C5B	0.0114 (15)	0.0102 (13)	0.0151 (14)	0.0002 (14)	−0.0001 (13)	−0.0042 (10)
C6B	0.0190 (16)	0.0067 (13)	0.0202 (14)	−0.0009 (14)	0.0039 (14)	−0.0019 (11)
C7B	0.0137 (15)	0.0125 (13)	0.0178 (14)	0.0023 (13)	0.0022 (14)	0.0019 (11)
C8B	0.0148 (15)	0.0104 (13)	0.0142 (13)	0.0035 (13)	−0.0013 (13)	0.0000 (11)
C9B	0.0123 (15)	0.0126 (14)	0.0124 (13)	−0.0020 (13)	0.0000 (13)	−0.0003 (10)
C10B	0.0155 (15)	0.0082 (13)	0.0131 (13)	0.0033 (14)	−0.0008 (13)	−0.0012 (10)
C11B	0.0161 (16)	0.0105 (13)	0.0125 (13)	0.0002 (13)	−0.0015 (13)	0.0032 (10)
C12B	0.0181 (17)	0.0073 (13)	0.0173 (14)	−0.0008 (13)	0.0001 (13)	−0.0024 (11)
C13B	0.0078 (14)	0.0156 (14)	0.0101 (13)	0.0014 (13)	−0.0008 (12)	−0.0015 (10)
C14B	0.0090 (15)	0.0132 (14)	0.0157 (14)	0.0013 (13)	−0.0007 (13)	0.0045 (11)
C15B	0.0186 (17)	0.0111 (13)	0.0132 (13)	0.0009 (14)	0.0009 (13)	0.0024 (10)
C16B	0.0173 (16)	0.0174 (14)	0.0109 (13)	0.0006 (14)	−0.0021 (12)	0.0025 (11)
C17B	0.0142 (16)	0.0133 (14)	0.0170 (14)	−0.0006 (14)	−0.0030 (13)	−0.0029 (11)
C18B	0.0209 (17)	0.0149 (16)	0.0167 (15)	0.0024 (14)	−0.0062 (13)	−0.0018 (13)
O19B	0.0324 (13)	0.0112 (11)	0.0125 (10)	0.0024 (9)	−0.0019 (10)	0.0015 (9)
O20B	0.0196 (12)	0.0227 (12)	0.0160 (11)	0.0050 (10)	−0.0058 (9)	−0.0068 (9)
C21B	0.0187 (16)	0.0165 (15)	0.0187 (15)	−0.0018 (15)	0.0013 (14)	−0.0054 (12)
O22B	0.0256 (12)	0.0097 (9)	0.0195 (10)	−0.0004 (10)	0.0039 (11)	−0.0013 (8)
O23B	0.0325 (13)	0.0138 (10)	0.0169 (10)	0.0022 (11)	0.0044 (11)	0.0042 (8)
O24B	0.0435 (14)	0.0147 (10)	0.0151 (10)	−0.0009 (12)	−0.0042 (11)	−0.0013 (8)
O25B	0.0178 (11)	0.0138 (10)	0.0118 (10)	0.0004 (9)	−0.0003 (8)	0.0019 (8)
C26B	0.0149 (17)	0.0177 (14)	0.0169 (14)	0.0017 (13)	−0.0008 (13)	−0.0007 (12)

### Geometric parameters (Å, º)

**Table:**

C1A—O19A	1.415 (3)	C1B—O19B	1.428 (3)
C1A—C10A	1.576 (4)	C1B—C10B	1.579 (4)
C1A—C2A	1.608 (4)	C1B—C2B	1.589 (4)
C1A—H1A	1.0000	C1B—H1B	1.0000
C2A—O25A	1.405 (3)	C2B—O25B	1.423 (3)
C2A—C3A	1.524 (4)	C2B—C3B	1.534 (4)
C2A—H2A	1.0000	C2B—H2B	1.0000
C3A—O20A	1.431 (3)	C3B—O20B	1.434 (3)
C3A—C4A	1.499 (4)	C3B—C4B	1.492 (4)
C3A—H3A	1.0000	C3B—H3B	1.0000
C4A—C21A	1.341 (4)	C4B—C21B	1.355 (4)
C4A—C5A	1.416 (3)	C4B—C5B	1.413 (3)
C5A—C6A	1.343 (3)	C5B—C6B	1.339 (3)
C5A—C10A	1.476 (4)	C5B—C10B	1.489 (3)
C6A—O22A	1.386 (3)	C6B—O22B	1.385 (3)
C6A—C7A	1.443 (4)	C6B—C7B	1.439 (4)
C7A—O23A	1.222 (3)	C7B—O23B	1.221 (3)
C7A—C8A	1.516 (3)	C7B—C8B	1.514 (4)
C8A—C14A	1.414 (3)	C8B—C14B	1.400 (4)
C8A—C9A	1.417 (3)	C8B—C9B	1.422 (3)
C9A—C11A	1.405 (4)	C9B—C11B	1.400 (3)
C9A—C10A	1.530 (3)	C9B—C10B	1.526 (3)
C10A—C18A	1.546 (4)	C10B—C18B	1.553 (4)
C11A—C12A	1.363 (3)	C11B—C12B	1.377 (3)
C11A—H11A	0.9500	C11B—H11B	0.9500
C12A—C13A	1.392 (3)	C12B—C13B	1.397 (3)
C12A—H12A	0.9500	C12B—H12B	0.9500
C13A—C14A	1.382 (4)	C13B—C14B	1.394 (3)
C13A—C17A	1.466 (4)	C13B—C17B	1.474 (4)
C14A—C15A	1.518 (3)	C14B—C15B	1.515 (3)
C15A—C16A	1.537 (3)	C15B—C16B	1.528 (4)
C15A—H15A	0.9900	C15B—H15C	0.9900
C15A—H15B	0.9900	C15B—H15D	0.9900
C16A—C17A	1.500 (4)	C16B—C17B	1.508 (4)
C16A—H16A	0.9900	C16B—H16C	0.9900
C16A—H16B	0.9900	C16B—H16D	0.9900
C17A—O24A	1.231 (3)	C17B—O24B	1.218 (3)
C18A—H18A	0.9800	C18B—H18D	0.9800
C18A—H18B	0.9800	C18B—H18E	0.9800
C18A—H18C	0.9800	C18B—H18F	0.9800
O19A—H19A	0.84 (3)	O19B—H19B	0.83 (3)
O20A—H20A	0.91 (3)	O20B—H20B	0.81 (3)
C21A—O22A	1.377 (3)	C21B—O22B	1.373 (3)
C21A—H21A	0.9500	C21B—H21B	0.9500
O25A—C26A	1.425 (4)	O25B—C26B	1.430 (3)
C26A—H26A	0.9800	C26B—H26D	0.9800
C26A—H26B	0.9800	C26B—H26E	0.9800
C26A—H26C	0.9800	C26B—H26F	0.9800
			
O19A—C1A—C10A	107.2 (2)	O19B—C1B—C10B	111.3 (2)
O19A—C1A—C2A	112.9 (2)	O19B—C1B—C2B	113.0 (2)
C10A—C1A—C2A	114.1 (2)	C10B—C1B—C2B	114.1 (2)
O19A—C1A—H1A	107.4	O19B—C1B—H1B	105.9
C10A—C1A—H1A	107.4	C10B—C1B—H1B	105.9
C2A—C1A—H1A	107.4	C2B—C1B—H1B	105.9
O25A—C2A—C3A	107.9 (2)	O25B—C2B—C3B	107.0 (2)
O25A—C2A—C1A	111.1 (2)	O25B—C2B—C1B	110.9 (2)
C3A—C2A—C1A	119.0 (2)	C3B—C2B—C1B	118.5 (2)
O25A—C2A—H2A	106.0	O25B—C2B—H2B	106.6
C3A—C2A—H2A	106.0	C3B—C2B—H2B	106.6
C1A—C2A—H2A	106.0	C1B—C2B—H2B	106.6
O20A—C3A—C4A	113.9 (2)	O20B—C3B—C4B	113.7 (2)
O20A—C3A—C2A	109.8 (2)	O20B—C3B—C2B	112.2 (2)
C4A—C3A—C2A	106.6 (2)	C4B—C3B—C2B	105.3 (2)
O20A—C3A—H3A	108.8	O20B—C3B—H3B	108.5
C4A—C3A—H3A	108.8	C4B—C3B—H3B	108.5
C2A—C3A—H3A	108.8	C2B—C3B—H3B	108.5
C21A—C4A—C5A	105.6 (2)	C21B—C4B—C5B	105.3 (2)
C21A—C4A—C3A	134.3 (3)	C21B—C4B—C3B	134.1 (3)
C5A—C4A—C3A	119.1 (2)	C5B—C4B—C3B	119.3 (2)
C6A—C5A—C4A	107.9 (2)	C6B—C5B—C4B	108.3 (2)
C6A—C5A—C10A	126.5 (2)	C6B—C5B—C10B	126.4 (2)
C4A—C5A—C10A	125.7 (2)	C4B—C5B—C10B	125.3 (2)
C5A—C6A—O22A	109.9 (2)	C5B—C6B—O22B	109.9 (2)
C5A—C6A—C7A	125.1 (2)	C5B—C6B—C7B	125.4 (2)
O22A—C6A—C7A	124.4 (2)	O22B—C6B—C7B	123.9 (2)
O23A—C7A—C6A	125.2 (2)	O23B—C7B—C6B	125.2 (2)
O23A—C7A—C8A	122.4 (2)	O23B—C7B—C8B	122.6 (2)
C6A—C7A—C8A	112.3 (2)	C6B—C7B—C8B	112.1 (2)
C14A—C8A—C9A	117.8 (2)	C14B—C8B—C9B	118.4 (2)
C14A—C8A—C7A	119.5 (2)	C14B—C8B—C7B	118.6 (2)
C9A—C8A—C7A	122.7 (2)	C9B—C8B—C7B	123.0 (2)
C11A—C9A—C8A	120.3 (2)	C11B—C9B—C8B	119.4 (2)
C11A—C9A—C10A	117.1 (2)	C11B—C9B—C10B	118.0 (2)
C8A—C9A—C10A	122.5 (2)	C8B—C9B—C10B	122.4 (2)
C5A—C10A—C9A	110.0 (2)	C5B—C10B—C9B	109.9 (2)
C5A—C10A—C18A	108.7 (2)	C5B—C10B—C18B	107.1 (2)
C9A—C10A—C18A	107.3 (2)	C9B—C10B—C18B	107.4 (2)
C5A—C10A—C1A	107.0 (2)	C5B—C10B—C1B	107.3 (2)
C9A—C10A—C1A	112.8 (2)	C9B—C10B—C1B	114.3 (2)
C18A—C10A—C1A	111.1 (2)	C18B—C10B—C1B	110.7 (2)
C12A—C11A—C9A	121.3 (2)	C12B—C11B—C9B	122.1 (2)
C12A—C11A—H11A	119.4	C12B—C11B—H11B	118.9
C9A—C11A—H11A	119.4	C9B—C11B—H11B	118.9
C11A—C12A—C13A	118.6 (2)	C11B—C12B—C13B	118.1 (2)
C11A—C12A—H12A	120.7	C11B—C12B—H12B	120.9
C13A—C12A—H12A	120.7	C13B—C12B—H12B	120.9
C14A—C13A—C12A	122.4 (2)	C14B—C13B—C12B	121.6 (2)
C14A—C13A—C17A	110.1 (2)	C14B—C13B—C17B	109.9 (2)
C12A—C13A—C17A	127.5 (2)	C12B—C13B—C17B	128.4 (2)
C13A—C14A—C8A	119.6 (2)	C13B—C14B—C8B	120.2 (2)
C13A—C14A—C15A	111.1 (2)	C13B—C14B—C15B	110.6 (2)
C8A—C14A—C15A	129.3 (2)	C8B—C14B—C15B	129.2 (2)
C14A—C15A—C16A	104.1 (2)	C14B—C15B—C16B	105.2 (2)
C14A—C15A—H15A	110.9	C14B—C15B—H15C	110.7
C16A—C15A—H15A	110.9	C16B—C15B—H15C	110.7
C14A—C15A—H15B	110.9	C14B—C15B—H15D	110.7
C16A—C15A—H15B	110.9	C16B—C15B—H15D	110.7
H15A—C15A—H15B	109.0	H15C—C15B—H15D	108.8
C17A—C16A—C15A	106.6 (2)	C17B—C16B—C15B	106.3 (2)
C17A—C16A—H16A	110.4	C17B—C16B—H16C	110.5
C15A—C16A—H16A	110.4	C15B—C16B—H16C	110.5
C17A—C16A—H16B	110.4	C17B—C16B—H16D	110.5
C15A—C16A—H16B	110.4	C15B—C16B—H16D	110.5
H16A—C16A—H16B	108.6	H16C—C16B—H16D	108.7
O24A—C17A—C13A	125.9 (2)	O24B—C17B—C13B	126.5 (2)
O24A—C17A—C16A	126.0 (2)	O24B—C17B—C16B	125.6 (2)
C13A—C17A—C16A	108.0 (2)	C13B—C17B—C16B	107.9 (2)
C10A—C18A—H18A	109.5	C10B—C18B—H18D	109.5
C10A—C18A—H18B	109.5	C10B—C18B—H18E	109.5
H18A—C18A—H18B	109.5	H18D—C18B—H18E	109.5
C10A—C18A—H18C	109.5	C10B—C18B—H18F	109.5
H18A—C18A—H18C	109.5	H18D—C18B—H18F	109.5
H18B—C18A—H18C	109.5	H18E—C18B—H18F	109.5
C1A—O19A—H19A	107 (2)	C1B—O19B—H19B	108 (2)
C3A—O20A—H20A	114 (2)	C3B—O20B—H20B	112 (2)
C4A—C21A—O22A	111.8 (2)	C4B—C21B—O22B	111.4 (2)
C4A—C21A—H21A	124.1	C4B—C21B—H21B	124.3
O22A—C21A—H21A	124.1	O22B—C21B—H21B	124.3
C21A—O22A—C6A	104.8 (2)	C21B—O22B—C6B	105.11 (19)
C2A—O25A—C26A	114.4 (2)	C2B—O25B—C26B	113.8 (2)
O25A—C26A—H26A	109.5	O25B—C26B—H26D	109.5
O25A—C26A—H26B	109.5	O25B—C26B—H26E	109.5
H26A—C26A—H26B	109.5	H26D—C26B—H26E	109.5
O25A—C26A—H26C	109.5	O25B—C26B—H26F	109.5
H26A—C26A—H26C	109.5	H26D—C26B—H26F	109.5
H26B—C26A—H26C	109.5	H26E—C26B—H26F	109.5

### Hydrogen-bond geometry (Å, º)

**Table:**

*D*—H···*A*	*D*—H	H···*A*	*D*···*A*	*D*—H···*A*
O19*A*—H19*A*···O19*B*^i^	0.85 (3)	2.08 (3)	2.886 (3)	160 (3)
O19*B*—H19*B*···O20*A*^ii^	0.83 (3)	2.30 (3)	3.002 (3)	143 (3)
O20*A*—H20*A*···O25*B*^iii^	0.91 (3)	1.85 (3)	2.717 (3)	160 (3)
O20*B*—H20*B*···O24*A*^iv^	0.81 (3)	2.05 (3)	2.842 (3)	166 (3)
C2*A*—H2*A*···O23*B*^v^	1.00	2.45	3.325 (3)	146
C2*B*—H2*B*···O23*A*	1.00	2.38	3.295 (3)	151
C11*A*—H11*A*···O19*A*	0.95	2.43	3.084 (3)	126
C11*B*—H11*B*···O19*B*	0.95	2.58	3.230 (3)	126
C18*A*—H18*A*···O20*A*	0.98	2.38	3.227 (3)	145
C18*B*—H18*D*···O20*B*	0.98	2.39	3.241 (4)	145
C21*A*—H21*A*···O24*A*^vi^	0.95	2.37	3.282 (3)	162
C21*B*—H21*B*···O24*B*^vii^	0.95	2.25	3.180 (3)	167

Symmetry codes: (i) −*x*+1, *y*−1/2, −*z*+1/2; (ii) −*x*+1/2, −*y*+1, *z*−1/2; (iii) −*x*+1/2, −*y*+1, *z*+1/2; (iv) *x*−1/2, −*y*+1/2, −*z*; (v) *x*+1, *y*, *z*; (vi) −*x*+1, *y*+1/2, −*z*+1/2; (vii) −*x*, *y*−1/2, −*z*+1/2.
